# The structural and functional brain networks that support human social networks

**DOI:** 10.1016/j.bbr.2018.02.019

**Published:** 2018-12-14

**Authors:** M.P. Noonan, R.B. Mars, J. Sallet, R.I.M. Dunbar, L.K. Fellows

**Affiliations:** a McGill University, Montreal Neurological Institute, 3801 Rue University, Montreal, H3A 2B4, Quebec, Canada; b Department of Experimental Psychology, University of Oxford, South Parks Road, Oxford, OX1 3UD, United Kingdom; c Wellcome Centre for Integrative Neuroimaging, Centre for Functional MRI of the Brain, Nuffield Department of Clinical Neurosciences, John Radcliffe Hospital, University of Oxford, Oxford, OX3 9DU, United Kingdom; d Donders Institute for Brain, Cognition and Behaviour, Radboud University Nijmegen, 6525 EZ, Nijmegen, The Netherlands

**Keywords:** ACC, anterior cingulate cortex, AF, arcuate fasciculus, ATC, anterior temporal complex, BET, brain extraction tool, CB, cingulum bundle, CC, corpus callosum, CSF, cerebral spinal fluid, DAS, left dorsal attention stream (r=right, l=left), DMN, default mode network (a=anterior, p=posterior), DMRI, diffusion magnetic resonance imaging, dlPFC, dorsolateral prefrontal cortex, EmC, Extreme Capsule, EPI, echo planer imaging, FA, fractional anisotropy, FNIRT, FMRIBs non-linear registration tool, FWHM, full width half maximum, Fx, Fornix, GLM, General linear model, GM, gray matter, ICA, independent component analysis, ILF, inferior longitudinal fasciculus, IFOF, Inferior Fronto-occipital Fasciculus, MELODIC, Multivariate Exploratory Linear Optimized Decomposition into Independent Components, MLF, middle longitudinal fasciculus, MNI, Montreal Neurological Institute, OR, Optic Radiations, PCC, posterior cingulate cortex, PreC, precuneus, ROI, region of interest, rsfMRI, resting-state functional magnetic resonance imaging, RSN, resting-state networks, SM, sensory motor, SNS, social network size, TBSS, tract based spatial statistics, UF, Uncinate Fasciculus, VBM, voxel based morphometry, vmPFC, ventromedial prefrontal cortex, WM, white matter, Social network size, Diffusion weighted imaging, Anterior cingulate cortex, Structural connectivity, Default mode network

## Abstract

Social skills rely on a specific set of cognitive processes, raising the possibility that individual differences in social networks are related to differences in specific brain structural and functional networks. Here, we tested this hypothesis with multimodality neuroimaging. With diffusion MRI (DMRI), we showed that differences in structural integrity of particular white matter (WM) tracts, including cingulum bundle, extreme capsule and arcuate fasciculus were associated with an individual’s social network size (SNS). A voxel-based morphology analysis demonstrated correlations between gray matter (GM) volume and SNS in limbic and temporal lobe regions. These structural changes co-occured with functional network differences. As a function of SNS, dorsomedial and dorsolateral prefrontal cortex showed altered resting-state functional connectivity with the default mode network (DMN). Finally, we integrated these three complementary methods, interrogating the relationship between social GM clusters and specific WM and resting-state networks (RSNs). Probabilistic tractography seeded in these GM nodes utilized the SNS-related WM pathways. Further, the spatial and functional overlap between the social GM clusters and the DMN was significantly closer than other control RSNs. These integrative analyses provide convergent evidence of the role of specific circuits in SNS, likely supporting the adaptive behavior necessary for success in extensive social environments.

## Highlights

• Fronto-temporal white matter tracts relate to social network size.
• Specific limbic and temporal regions are larger in people with larger social networks.
• Functional coupling of the default mode network varies with social network size.

## 1 Introduction

Humans are inherently social creatures. We have the ability not only to tolerate conspecifics, but also to closely cooperate with them, through behaviours thought to be distinctively human, including our extended use of culture and language [1]. The advanced social abilities of humans, and to a lesser extent of other primates, have been related to the large increase in brain size in these species. The ratio of brain size to body size between species correlates with the number of individuals in social groups, a variable that indexes the social complexity of a species’ life [2,3]. Better social abilities may have helped primates deal with predators and, when group sizes became larger, cooperate with conspecifics.

Early lesion work in humans and monkeys emphasised the contribution of the prefrontal cortex [4,5] and the anterior cingulate cortex in particular [6,7] to social behaviour. Yet socio-cognitive capacities can also be notably impaired when brain damage is diffuse or multifocal [8,9], or when multiple systems are affected, as happens in certain psychiatric conditions [10,11] and neurodegenerative diseases [12]. This argues that more extensive brain networks are engaged in supporting various aspects of social behaviour, a claim also supported by converging network-level neuroimaging evidence [13,14] and human fMRI studies [[15], [16], [17], [18]].

Taking advantages of advances in MRI analytics, which now provide tools to study brain-behaviour relationships at the level of circuits and networks, a series of macaque imaging studies investigated network-level causal changes associated with increased socio-cognitive pressures [19]. With the individual’s social network size (SNS; a summary indicator of social abilities) under experimenter control, changes were observed in gray matter (GM) and inter-regional functional coupling: more GM volume in medial prefrontal cortex and connected regions such as the amygdala and middle part of the superior temporal sulcus (mSTS, [19]) was reported in animals living in larger social groups. Further, functional coupling between the mSTS and the ACC [19], as well as between the ACC and the Default Mode Network (DMN) [20] varied as a function of SNS.

The present study set out to test predictions derived from the findings in macaque monkeys [19,20] and identify networks with comparative functional or structural homology in a healthy human sample. Unlike previous work on the brain basis of social networks in humans [[21], [22], [23], [24], [25], [26], [27]], here we sought converging evidence across multiple imaging modalities, diffusion MRI (DMRI), structural, and resting-state functional MRI (rsfMRI). The dependent and independent measures, and analytical techniques we used were equivalent to those used in the macaque work. We predicted that fronto-temporal WM, GM and functional network differences would each relate to SNS in humans. We then integrated across these methodologies, to find the consistent structural and functional links that underlie brain network organisation [e.g. 28]. We reasoned that effects that replicated across imaging modalities would be the most compelling, and would be a useful step towards a mechanistic understanding of the neural substrates of the human social behaviour.

Specifically, the first analysis investigated structural WM differences associated with SNS using DMRI. Next, we used voxel-based morphology to define brain regions where GM volume correlated with SNS. Given those results, and the key role of ACC in the macaque work on SNS, dual regression analysis was then used to examine the functional interactions between the ACC and the default mode network (DMN). Lastly, we performed cross-modal validations by integrating these three complementary imaging methods, to test whether these individual observations could be related in network terms, interrogating the relationship between SNS-associated GM clusters and specific WM tracts, and RSNs.

## 2 Materials and methods

### 2.1 Subjects

18 right-handed people (11 women) recruited from the Montreal community participated in the present imaging experiment. Participants had a mean age (and standard deviation) of 51.9 yr (15.3). All had normal or corrected-to-normal vision and indicated no history of psychiatric or neurological disease. All subjects scored within the normal range on screening tests of cognitive ability (>26 in the Montreal Cognitive Assessment, a screening tool for mild cognitive impairment in older people [29]) and depression (<16 in the Beck Depression Inventory (II)). All subjects gave informed consent to participate in the investigation, which had been approved by the Research Ethics Board of the Montreal Neurological Institute.

### 2.2 Social questionnaires

Information about social network size was obtained using a written questionnaire. Following prior reports [21,30], participants were asked to list the initials of every individual with whom they had personal contact or communication over the previous 7 and 30 days. The instructions were: ‘In the spaces below, please list the INITIALS of everyone with whom you had some kind of social contact (a) during the last 7 days and (b) during the rest of the last month (i.e. approx. 30 days). Contact means some form of interaction, including face-to-face, phone call, email or text-messaging, or a letter. Please DO NOT INCLUDE people whom you contacted for professional reasons (e.g. your doctor, lawyer, hairdresser, priest, employer or supervisor, plumber or DIY consultant etc.) UNLESS you considered that interaction to have been of a mainly SOCIAL nature at the time. You can look at a list of names in your phone/address book if this helps.’ These metrics have been shown to scale up, serving as reliable indicators of the whole social network, conventionally defined as social contacts in the past year [31].

### 2.3 Image acquisition

Participants lay supine in the scanner and cushions were used to reduce head motion. All images were acquired on a 1.5T Siemens MR scanner at the McConnell Brain Imaging Centre at the Montreal Neurological Institute, McGill University. BOLD fMRI data were acquired by using echo planar imaging (EPI) (36 × 4 mm thick axial slices with a base resolution of 64mm, field of view 256 × 256 × 144 mm^3^, giving a voxel size of 4 × 4 × 4 mm, repetition time = 2.8s, 153 volumes, echo time = 50 ms, and flip angle = 90°). The EPI scanning sequence lasted 7 min 20 s and subjects were instructed to keep their eyes closed, think of nothing and not fall asleep. A T1-weighted anatomical image was acquired for each subject (repetition time = 2800 ms, echo time = 4.12 ms, and flip angle = 15°, giving a voxel size of 1 × 1 × 1 mm). Diffusion MRI (DMRI) data were also acquired from 17 of the same subjects described above, with the same scanner. A technical issue meant it was not possible to collect the DMRI in the 18th subject. DMRI data were acquired using echo planar imaging (75 slices, 2 mm thick axial slices; field of view, 256 × 256 × 150 mm; giving a voxel size of 2 × 2 × 2 mm). Diffusion weighting was isotropically distributed along 99 directions using a B value of 1000 mm^2^. 10 volumes with no diffusion weighting were acquired throughout the acquisition. The total scan time for the DMRI protocol was 20.21 min.

### 2.4 Data analysis

**Fig. 1:**
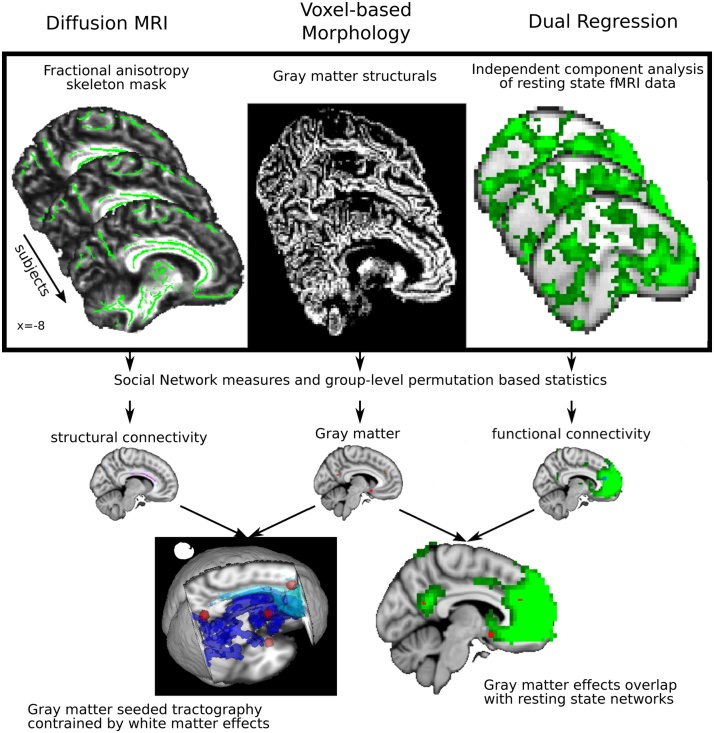
Schematic depicts the overall methodological approach of the study. In the top panel, illustrated for 3 subjects, from left to right we analyzed DMRI, structural and rsfMRI data. We related differences in WM (purple-turquoise), GM (yellow) and functional coupling within RSNs (blue), to individual measures of SNS (mid panel). These techniques were integrated as illustrated by the converging arrows on the lower panel. We used WM tracts, where fractional anisotropy covaried with SNS (dark blue) to constrain probabilistic tractography analyses (turquoise) seeded from social GM clusters (red, bottom left). We also compared the spatial topography of RNSs (green) with social GM clusters (red, bottom right). (For interpretation of the references to colour in this figure legend, the reader is referred to the web version of this article).

Fig. 1 outlines the analyses, which aimed to build a convergent case for the brain networks related to SNS. Separately, we first identified [1] WM, [2] GM and [3] RSNs that differ as a function of SNS. Importantly, we used the same design matrix to analyse the data from the three imaging techniques, allowing the findings to be directly integrated in the next step of the analysis where we investigated whether the results obtained in the single modalities were indicative of changes within the same neural networks. To this end, we took the GM clusters associated with SNS as the starting point, and interrogated their relationships with specific WM and RSNs. We seeded probabilistic tractography analyses in these GM clusters and constrained the tracts to the WM tracts separately associated with SNS. We also examined the spatial and functional overlap between GM clusters and RSNs.

#### 2.4.1 Preprocessing

Data were analyzed using tools from the FMRIB Software Library

(www.fmrib.ox.ac.uk/fsl). All structural and EPI images were converted to NIFTI and skull stripped with BET; where appropriate this stage was corrected by hand. All brain images are shown in the radiological convention throughout the paper.

#### 2.4.2 White matter correlates of SNS

DMRI data were preprocessed using tools from FDT (for FMRIB’s Diffusion Toolbox; part of FSL 4.1). Eddy-current distortions were corrected using affine registration of all volumes to a target volume with no diffusion weighting.

For all subsequent analyses, tracts were identified on the basis of their location, routes, and cortical projections areas as discussed in the human brain connectivity atlas of Catani and De Schotten [32], NatBrainLab online catalogue (http://www.natbrainlab.co.uk/atlas-maps), JHU White-Matter Tractography Atlas [33] and the atlases of white matter tracing studies in the macaque of Schmahmann and Pandya [34].

##### 2.4.2.1 Tract based spatial statistics

As depicted in Fig. 1 (upper left panel) we assessed correlations between WM integrity and SNS with the FSL Tract-Based Spatial Statistics (TBSS) processing pipeline [35 http://fsl.fmrib.ox.ac.uk/fsl/fslwiki/TBSS]. Specifically, the preprocessed data were subjected to DTIFIT, an analysis step which fits a diffusion tensor model at each voxel in order to generate a 3D fractional anisotropy image for each subject. This image was registered to the FMRIB58FA standard brain before a study specific skeletonised FA template was generated and thresholded at 0.2. All subjects’ skeletonized FA images were concatenated and the resulting 4D image was subjected to voxel-wise cross-subject statistics using non-parametric permutation testing [36] with Randomise [37]. The GLM included factors of the demeaned size of the social network as well as the confound regressors of age, sex and number of years in education. These confound regressors account for potential age-related atrophy, structural and functional differences related to gender, and general intellectual abilities that may affect SNS. All reported statistics were found to survive cluster correction for multiple comparisons (p < 0.05) with threshold free cluster enhancement methods [38].

While we acknowledge no formal interpretation of intra-cluster features can be made from a cluster-level inference, we illustrate the relationships between SNS and the mean FA value extracted from six ROI sections taken from within the large significant cluster. The Matlab Regstats function was used to calculate the residual mean FA effect size from 20 contiguous voxels in each ROI section and SNS after controlling for confounding effects of age, gender and number of years in education. With reference to white matter atlases, six ROIs were chosen from sections of tracts that were visually identified as right and left cingulum bundle (CB, MNI: 10, 7, 33 and −7, 15, 28) and extreme capsule (EmC, MNI: 33, 5, 5 and −34, −15, −3), right arcuate fasciculus (AF, MNI: 43, −38, 32) and corpus callosum (CC, MNI: 0, −2, 25).

To ensure results could not be explained by head motion or total intracranial volume, the residual effect size, after these additional confound regressors were accounted for, was correlated with SNS. To examine reliability and determine if a single outlier was driving effects, we performed a leave-one-out analysis using a jack-knife procedure. For the size of the sample, we computed Pearson’s correlation coefficients of the mean FA of the whole WM cluster while each subject was, in turn, left out of the analysis.

##### 2.4.2.2 White matter seeded tractography

The six sections illustrating the FA SNS correlations were relatively unambiguously associated with a particular WM tract. However, according to reference atlases, other sections of FA effects could have been contiguous with the CB, EmC or AF or could have reflected a number of alternative tracts including middle or lateral longitudinal fasciculus (MLF, ILF). Therefore, to confirm our visual identification of WM tracts we performed two complimentary probabilistic tractography analyses seeded from the TBSS effects.

First, in a targeted hypothesis driven analysis, the right hemisphere ROI sections for the CB, AF and EmC described above were dilated using fslmaths, registered to individual subject space and used as seed masks for probabilistic tractography. Voxel-wise estimates of the fiber orientation distribution were calculated using Bedpostx, limited to estimating two fiber orientations at each voxel, because of the b value and number of gradient orientations in the diffusion data [39 http://fsl.fmrib.ox.ac.uk/fsl/fslwiki/FDT]. Probabilistic tractography was run for each subject, using a model accounting for multiple fiber orientations in each voxel. Five thousand sample streamlines were seeded from each voxel within each individual’s seed mask. The tractography algorithm parameters used were a maximum of 2000 steps; step size of 0.5 mm and a curvature threshold of 0.2. Each streamline followed local orientations sampled from the posterior distribution given by BedpostX, as described previously. In the first analysis a visitation map or tractogram was constructed for each individual while the second analysis resulted in a subject-specific seed x mask tract matrix, but both represented connectivity distribution from the seed mask. These connectivity distribution values were log transformed, normalized by dividing by the maximum tracts identified for each subject, and thresholded at 0.8 and binarised [40]. In doing so we sought only the top 20% of tracts emanating from the seed mask. Finally, for visualization the tracts were summed across subjects, registered to MNI space and are illustrated thresholded at more then 50% of subjects.

The second analysis used a novel data driven approach in which we combined tractography with principle component analysis in order to identify the dominant WM tracts that overlap with the whole FA WM effects. Probabilistic tractography was seeded from all significant TBSS voxels within MNI space (downsampled to 2 mm). Voxels in the corpus callosum, identified from the JHU White-Matter Tractography Atlas, were excluded from the seed mask as tractography seeded from corpus callosum regions dominate the subsequent principle component analysis. BedpostX, probabilistic tractography and thresholding parameters were identical to those described for the first analysis. Connectivity was quantified between each seed voxel and a whole brain target mask. The resulting connectivity matrices (a 2D seed x target mask tractogram) were concatenated across subjects and subjected to singular value decomposition (SVD, Matlab). SVD identifies large-scale patterns of variance in the population of subjects and seed masks. The positive and negative contrasts from the top 20 components were then compared to seven pre-defined WM tracts from the NatBrainLab catalogue. The WM tracts selected were the Arcuate Fasciculus (AF), Cingulum Bundle (CB), Fornix (Fx), Inferior Longitudinal Fasciculus (ILF), Inferior Fronto-occipital Fasciculus (IFOF), Optic Radiations (OR) and Uncinate Fasciculus (UF). The distinction between the IFOF and EmC has been questioned, so we refer to this tract in these analyses as EmC/IFOF. The final step quantified the percentage of spatial overlap for each component and each tract, comparing component coverage across all pre-defined WM tracts in an across-component one-way ANOVA.

#### 2.4.3 Gray matter correlates of SNS

We used Voxel-Based morphometry [41 http://fsl.fmrib.ox.ac.uk/fsl/fslviki/FSLVBM] to identify areas of GM where volume correlated with SNS (upper centre panel of Fig. 1). The skull stripped T1-weighted structural images were individually segmented into gray matter (GM), WM and cerebral spinal fluid (CSF) before being affine-registered to the GM ICBM-152 template using FLIRT [42] followed by nonlinear registration using FMRIB’s Nonlinear Image Registration Tool (FNIRT) [43]. The resulting images were averaged to create a study specific template to which the native GM images were then non-linearly re-registered and concatenated into a 4D image. The registered partial volume images were then modulated (to correct for local expansion or contraction) by dividing by the Jacobian of the warp field. The modulated segmented images were then smoothed with an isotropic Gaussian kernel with a sigma of 4 mm.

The resulting Jacobian 4D image was then used within a GLM analysis which included factors of the size of the social network, sex, age and number of years in education, which was implemented using permutation-based non-parametric testing with Randomise (n = 5000). First, we report only regions that bilaterally survive. This approach was proposed by the originators of MRI voxel-based GM analyses [44], as cluster corrected measures can be more prone to Type II errors (false negatives) [45]. The approach of finding similar effects in bilaterally symmetrical structures was used in some of the earliest human GM analyses by some of these investigators [46], as well as in recent work [47,48]. The bilaterality premise rests on the assumption that if a given statistical effect had a chance of occurrence of p ≤ 0.01 in one brain area under the null hypothesis, then it has the chance of occurring in the same area in both hemispheres with the square of this probability (i.e., p ≤ 0.0001) [49]. This method was implemented by thresholding and binarising the uncorrected p-map image at p ≤ 0.01, flipping the p-map image along the x dimension and multiplying the two images. We applied a spatial extent threshold of >40 voxels (each voxel being 2 mm^3^, therefore spatial extent exceeded 320 mm^3^). Second, in addition to the whole brain approach, we also adopted a hypothesis-driven ROI approach. We examine effects in predefined ROIs using a threshold of p < 0.05 and correction for multiple comparisons across all voxels in the ROI. As noted above, there are a priori reasons for thinking that SNS may be associated with the ACC. The clusters reported by Sallet, Mars [19] and Mars, Neubert [20] as structurally and functionally varying with macaque SNS fall within monkey areas 24ab and area 32. We therefore used the human structural homologues of these regions, defined by Neubert et al [50] as our ROI (See Neubert’s Cingulate Orbito-frontal Parcellation http://www.rbmars.dds.nl/CBPatlases.htm). First, we calculated the centre of gravity from a combined mask of bilateral areas 24ab and 32. We then placed a mask with a radius of 7.5 voxels at these coordinates. Non-GM and non-cingulate voxels were removed. Finally, as the resulting ROI sphere crossed the hemisphere, it was divided along the mid-sagittal section into two lateralized hemisphere ROIs (each ∼8960 mm^3^).

For illustrative purposes, we show the relationships between SNS and the mean GM effect size extracted from a 64 mm^3^ ROI placed at the centres of gravity of the regions identified as having a significant relationship with SNS. The Matlab Regstats function was used to calculate the residual deformation based morphology effect size and SNS after controlling for confounding effects of age, gender and number of years in education. In a separate analysis, head motion and total intracranial volume were also include as confound regressors. Again these effects were validated using a leave-one-out analysis jack-knife procedure.

#### 2.4.4 Resting state functional connectivity correlates of SNS

Each subject’s individual functional EPI data were first preprocessed using Multivariate Exploratory Linear Optimized Decomposition into Independent Components (MELODIC). Components that were clearly caused by head motion or spikes were removed.

Resting state functional connectivity was assessed using the Dual Regression technique [51 http://fsl.fmrib.ox.ac.uk/fsl/fslwki/DualRegression]. This three-step method allows for voxel-wise comparisons of resting functional connectivity. First, all subjects’ denoised rsfMRI data is collectively motion corrected, spatially smoothed (using a Gaussian kernel of full-width at halfmaximum (FWHM) of 6 mm) and high-pass temporally filtered to 150 s (0.007 Hz). Individual fMRI volumes were registered to the individual’s structural scan and standard space images using FNIRT. Preprocessed functional data containing 154 time points for each subject were temporally concatenated across subjects to create a single group 4D FMRI data set. This concatenated group data set is then decomposed using independent component analysis (ICA). ICA is used to identify large-scale patterns of functional connectivity in the population of subjects. In this analysis, the data set was decomposed into 25 components, in which the model order was estimated using the Laplace approximation to the Bayesian evidence for a probabilistic principal component model. We can select specific RSNs, defined by ICA, by spatial correlation against a set of previously defined networks. Based on previous work [20] we focused on the Default Mode Network (DMN) which here decomposed into an anterior (aDMN) and posterior (pDMN) component. We also used three other RSNs as control networks: sensory-motor and left, and right, dorsal attention stream. For these control RSNs, we predicted no changes in intra-network coupling as a function of SNS.

The second step uses the dual-regression approach to identify, within each subject’s fMRI data set, subject-specific temporal dynamics and associated DMN spatial maps. This involves (i) using the full set of group-ICA spatial maps in a linear model fit (spatial regression) against the separate fMRI data sets, resulting in matrices describing temporal dynamics for each component and subject, and (ii) using these time-course matrices in a linear model fit (temporal regression) against the associated fMRI data set to estimate subject-specific spatial maps. The third and final step concatenates the DMN component map across subjects into single 4D files (1 per original ICA map, with the fourth dimension being subject identification) and uses non-parametric permutation testing (Randomise n = 5000, with cluster-based thresholding c = 3.1, significance p < 0.05) to examine voxel-wise statistically significant between-subject differences [36] and results in spatial maps characterizing the between-subject differences. The GLM included factors of the size of the social network as well as the confound regressors of age, sex and number of years in education. While no cluster survives correction for multiple comparisons at the whole brain level, based on our a priori hypotheses concerning the contribution of the prefrontal cortex to the DMN we corrected for multiple comparisons within two small volumes of interest. These ROIs were [1] anatomical masks based on the centre of gravity of areas 24ab and area 32 (same as VBM analysis) and [2] the thresholded (p < 0.05, clusters greater than 100 voxels) group-ICA DMN component (aDMN 203,328 mm^3^ and pDMN 123,712 mm^3^). For the control RSNs, we performed the equivalent small volume correction over the thresholded group-ICA network (sensory-motor ROI = 142,016 mm^3^, left dorsal attention stream ROI = 205,312 mm^3^, right dorsal attention stream ROI = 246,272 mm^3^). In a control analysis, we confirmed GM partial volume effects did not drive the Dual Regression results by including the 4D jacobian image file as a voxel-dependent EV in the FSL GLM design matrix, akin to biological parametric mapping [52,53].

For illustrative purposes in scatter plots, we show the relationships between SNS and the mean z-value of the individual’s dual regression component maps extracted from 64 mm^3^ ROIs encapsulating the significant clusters (p < 0.05). The Matlab Regstats function was used to calculate the residual resting state effect size and SNS after controlling for confounding effects of age, gender and number of years in education. In a separate analysis, head motion and total intracranial volume were also include as confound regressors. Further, we performed a leave-one-out analysis with a jack-knife procedure.

#### 2.4.5 Network connections

In two final analyses, we integrated across the imaging methodologies to relate the individual findings to each other, within an overall network framework. We tested whether GM regions identified by the VBM analysis as larger in more social individuals are associated with the structural and functional networks identified by TBSS and Dual Regression, themselves also different in subjects with larger social groups.

##### 2.4.5.1 Gray matter seeded tractography

**Fig. 2:**
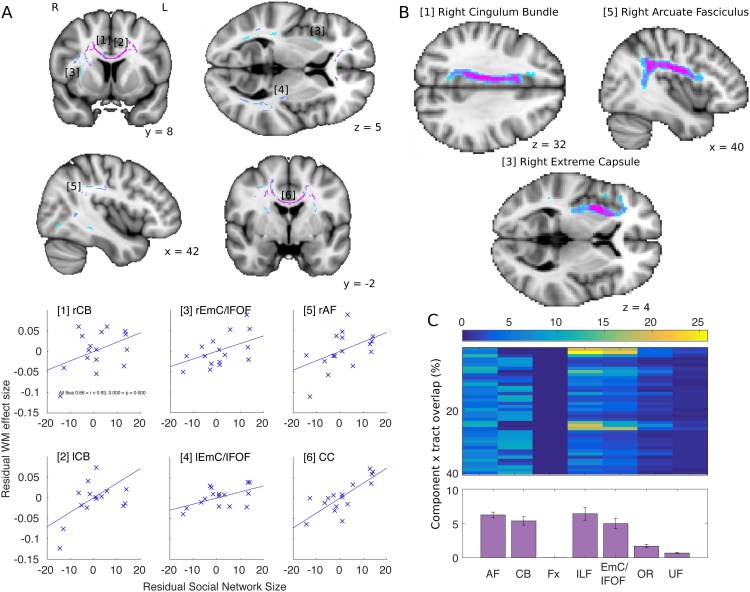
[A] TBSS results showing fiber pathways (purple-turquoise colors tfce corrected p < 0.05) in which FA correlates with SNS. For illustrative purposes we show the relationships between residual SNS and the mean FA value extracted from ROIs masks positioned in identifiable white matter tracts. [B] Tractography results from 3 probablistic tractography investigations seeded in the subsections of white matter identified in A; [1] right cingulum bundle, [3] right extreme capsule and [5] right arcute fasciculus. Visual thresholds set ≥9–17 subjects. Intensity from turquoise to purple represents number of subject’s with overlapping tracts. Images are in radiological convention. [C] Percentage overlap between components from the tractography decomposition analysis and published WM tracts from the NatBrainLab. Bar plot shows mean percentage overlap and between component standard error. Abbreviations: CB cingulum bundle, EmC/lFOF extreme capsule / inferior fronto-occipital fasciculus, AF arcute fasciculus, CC corpus callosum, Fx fornix, ILF inferior longitudinal fasciculus, OR optic radiations, UF uncinate fasciculus. (For interpretation of the references to colour in this figure legend, the reader is referred to the web version of this article).

To assess connectivity between the gray and white matter structures identified in the single modality analyses, we seeded five tractography analyses in each of the clusters where GM volume correlated with SNS was identified in the VBM analysis. Using the same tractography protocol described above, a single waypoint image was made by dilating the significant TBSS clusters (any voxel surviving p < 0.05, corrected for multiple comparisons; Fig. 2). Analogous to the analyses described above, each individual’s tractogram was log transformed, normalized, thresholded at 0.8, binarised and registered to MNI space. Again, to identify the resulting tracts we calculated the percentage of spatial overlap for each subject’s seed-specific tractogram and 7 NatBrainLab WM tracts. We compared coverage in a 5 (GM cluster; lATC, rATC, PCC/PreC, ACC, vmPFC) × 7 (Tracts; AF, CB, Fx, ILF, IFOF, OR and UF) repeated measures ANOVA. To perform follow-up comparisons for each tract we averaged the percentage overlap across GM clusters and compared across tracts with paired-samples t-tests.

We also quantified the structural connectivity between each VBM cluster (Fig. 1, lower left panel). The same protocol was used as described directly above but now in each analysis the four non-seed regions acted as classification targets and again pathways were constrained to the TBSS cluster effects. Probtrackx quantifies the connectivity values between the GM seed mask and the GM target mask, with the tracts only counted if they pass through the WM waypoints. The value of each voxel within the seed mask is the number of samples seeded from that voxel reaching the relevant target mask. We calculated the median connectivity values across voxels for each subject and normalised by the product of the size of the seed and target mask. For each GM cluster seed we then averaged each of the five seed-target analyses (eg seed [ACC]-to-targets [(rATC + lATC + PCC + vmPFC)/4]). A one-way repeated measures ANOVA across seed regions and post hoc t-tests isolated regions with greater connectivity within the network.

##### 2.4.5.2 Gray matter relationship with resting state networks

We examined the structural and functional relationship between GM differences and RSNs. First, we calculated the percentage of voxel-wise spatial overlap between the five group-level social GM clusters and each individual subject’s DMN components, normalized by the total size of the component. We also calculated this measure for control RSNs that we hypothesized would be less involved in social cognition (sensory-motor and the right and left dorsal attention streams). We compared the results in a one-way repeated measures ANOVA with 5 levels of RSNs and follow-up post-hoc t-tests.

Second, we examined the degree of functional correlation between the VBM clusters and ICA components. For each subject, we extracted the raw resting-state time courses from five ROIs based on the coordinates of the centre of gravity of the GM clusters (spherical masks radius = 3). For each subject, we regressed the timecourses of each RSN, as well as the whole brain time series, against each mask. The RSNs compete to explain the variance in the GM cluster ROI time course. Beta values are compared in a 5 (GM cluster; lATC, rATC, PCC/PreC, lACC, vmPFC) × 5 (RNS; SM, rDAS, aDMN, lDAS, pDMN) repeated measures ANOVA. Post-hoc follow up one-sample and paired t tests were run on the averaged beta values across all GM clusters i.e. the variance explained by each RNS time series, averaged across all GM clusters.

## 3 Results

### 3.1 Social network size correlates with FA in specific fronto-temporal white matter tracts

We first investigated whether there is a relationship between SNS and the structural WM connections between cortical areas. Medial frontal and temporal cortex are associated with SNS [19,20]. We therefore hypothesized that the integrity of fronto-temporal white matter tracts connecting these regions would be greater in individuals’ with larger SNS. Using tract-based spatial statistics, we tested whether functional anisotropy (FA) of WM voxels was predicted by SNS across subjects. SNS was calculated as the number of individuals with whom the subject had some form of social contact in the last 30 days [cf. 54], an established index known to correlate with total SNS [31]. We also performed probabilistic tractography from these voxels to identify the larger tracts to which they belong.

A large cluster-corrected swath of white matter, encompassing several WM tracts known to connect temporal and frontal cortical areas, showed a positive correlation between FA and SNS. Within this contiguous cluster, four anatomically specific WM pathways were clearly identifiable. Fig. 2A(1–6) illustrates the relationship between individual SNS and FA in different probe sections taken from the larger cluster. Tractography seeded from the probe section allows visualization of the full tract to support identification (Fig. 2B). Seeding tractography from the right hemisphere of the two CB sections (Fig. 2A_(1,2)_) confirms this tract running medially anterior-posterior through the brain. The CB connects, among others, the ACC and posterior cingulate cortex [55,56]. The posterior portion of the WM cluster (Fig. 2A_(5)_) is likely to belong to a curving tract connecting parts of the posterior cortex with frontal cortical areas. Based on the morphometry and projection areas of this tract, we attribute this part of the WM to the AF (Fig. 2B). Tractography seeded from the right hemisphere section of WM along the temporal extent of the cluster, continuing into the medial part of the frontal cortex (Fig. 2A _(3,4)_), demonstrates a tract identified by different authors using different nomenclatures (Fig. 2B). The NatBrainLab and De Schotten and colleagues [32] refer to it as the inferior fronto-occipital fascicle (IFOF), but Pandya and colleagues [34,57] have argued that this tract should be referred to as the extreme capsule (EmC).

Apart from the connections between temporal and frontal cortex, correlation with SNS was also observed throughout the corpus callosum (Fig. 2_(6)_). These effects appear not to be specific to anatomically defined DTI tract-based parcellations [58]. We direct readers to http://datasharedrive.blogspot.co.uk/2015/05/brain-networks-for-social-networks.html to view the complete statistical image from this analysis. Importantly, the TBSS effects were not driven by a single outlier subject. A leave-one-out analysis of the mean FA across the whole WM cluster showed the results were robust, with all correlations remaining significant after the removal of any single subject (0.86 < r < 0.93). Further, the effects cannot be explained by head movement or total intracranial volume, with the cluster remaining significantly correlated with SNS after the variance explained by these confound regressors was removed (FD r = 0.94, p < 0.0001, 0.89, TIV p < 0.0001).

We confirmed our interpretation of the WM effects with a data-driven analysis that combined probabilistic tractography and principal component analysis. This analysis aims to identify the dominant WM tracts that overlap with the whole FA WM effects. For each subject, probablistic tractography was seeded from each voxel where FA was positively correlated with SNS and estimated connectivity values to any other brain voxel. The resulting connectivity matrixes were concatenated across subjects and single vector decomposition analysis used principles of dimensionality reduction to identify the tracts that explain most variance in the connectivity matrixes. We isolated the top 20 of these components (each with a positive and negative contrast) and show the percentage of spatial overlap between these components and seven independently pre-defined WM tracts from the NatBrainLab (Fig. 2C). Complementing our visual inspection of the TBSS effects, the components overlap most with AF, CB and EmC/IFOF. This analysis also identified the ILF. While the EmC/IFOF and the ILF tracts defined by NatBrainLab share common space, it is clear that the components overlap with independent WM in both tracts.

Differences in percentage overlap between components and predefined WM tracts were analysed in a one way ANOVA of the mean percentage overlap across all components, revealing a main effect of Tract (F_6,234_ = 27.10, p < 0.001). Follow-up paired comparisons suggest that the tracts dominating the PCA analysis, AF, CB, EmC/IFOF and ILF, fail to do so differentially (AF vs CB: t_39_ = 1.02, p = 0.316, AF vs ILF: t_39_ = −0.194, p = 0.848, AF vs EmC/IFOF: t_39_ = 1.58, p = 0.122, CB vs ILF: t_39_ = −0.72, p = 0.478, CB vs EmC/IFOF: t_39_ = 0.31, p = 0.757). All other comparisons are significant (t > 4.36).

### 3.2 Human social network size correlates positively with regions implicated in social behaviour

**Fig. 3:**
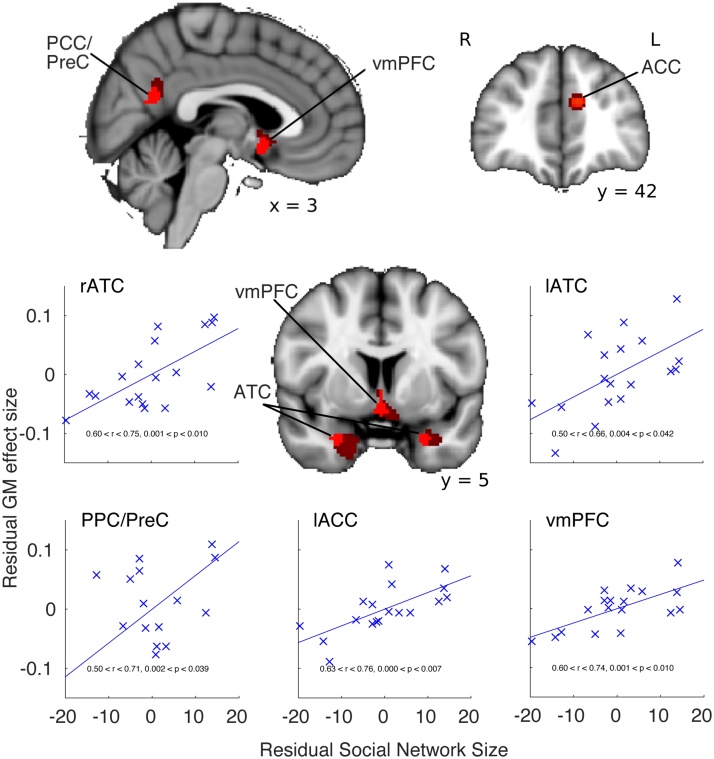
Linear positive correlations between gray matter volume and social network size. Prominent effects are evident in regions involved in social cognition, mentalising and face processing. These include vmPFC, anterior temporal cortex and PCC/precuneus (red p < 0.0001, bilateral uncorrected, cluster extent > 500 mm^3^) and the ACC (SVC, p < 0.05). Images in radiological convention. Mean gray matter from an ROI (64 mm^3^) placed at the centre of gravity of clusters is extracted for each subject. This approach is used even when the gray matter cluster crosses the hemispheric boundary. For illustration, dark red p-map illustrates non-lateralised effects p < 0.01 uncorrected, cluster extent >800 mm^3^). Scatter plots illustrate the residual gray matter effects against the residual of SNS, accounting for age, gender and years of education. Abbreviations: ATC anterior temporal cortex, PPC/PreC posterior parietal cortex / precuneus, ACC, anterior cingulate cortex, vmPFC ventromedial prefrontal cortex. (For interpretation of the references to colour in this figure legend, the reader is referred to the web version of this article).

The previous analysis suggested that a specific network of WM known to connect frontal and temporal cortex has higher integrity in subjects with larger social networks. Explicitly motivated by prior work in the macaque [19], we next performed a voxel based morphology (VBM) analysis to identify this GM network in this sample and investigate whether differences in local GM volume are similarly associated with SNS. Seeking areas that were present in both hemispheres, across the bilateral and ROI approach, we identified four regions that showed a positive correlation with the size of an individual’s social network (Fig. 3). Subcallosal parts of the ventromedial prefrontal cortex (vmPFC) including cingulate gyrus and extending to the septum, anterior temporal cortex (ATC, composing the amygdaloid complex/temporal pole), and the border of posterior cingulate cortex and precuneus (PCC/PreC) were evident in both hemispheres (p ≤ 0.0001, bilateral uncorrected, cluster extent >500 mm^3^).

An ROI-based analysis grounded in a priori predictions of the involvement of the ACC in sociocognitive behaviour [19] revealed a localised cluster in the left ACC that survived small volume correction (SVC) for multiple comparisons (p < 0.05). A right hemisphere ACC cluster was also identifiable, offset posteriorly by 14 mm, but did not survive cluster correction (p = 0.15, cluster extent = 128 mm^3^, MNI 8 28 22). See Table 1 details full MNI coordinates and cluster extents and http://datasharedrive.blogspot.co.uk/2015/05/brain-networks-for-social-networks.html to view the complete uncorrected statistical image from this analysis.

**Table 1:** MNI centre of gravity coordinates and cluster extent of gray matter volume significantly correlated with SNS in humans.

Number of significant voxels (2 mm^3^)	X	Y	Z	Region
49	0	−58	22	Bilateral Posterior Cingulate/ PreCuneus
47	0	10	−8	Bilateral ventromedial PFC
82	26	2	−26	Right Anterior temporal cortex
82	−26	2	−26	Left Anterior temporal cortex
42	−10	42	22	Left Anterior cingulate cortex

Again, a leave-one-out analysis on the GM effect for the six clusters showed that these effects were not driven by a single outlier subject (0.5 < r < 0.76). Further, the results were robust to head movement or total intracranial volume, with the effects remaining significant after removal of the variance explained by these confound regressors (all FD 0.59 < r < 0.69, 0.002 < p < 0.0094, TIV 0.5678 < r < 0.6682, 0.002n< p < 0.014).

### 3.3 Social network size modulates the functional coupling of dorsolateral and dorsomedial frontal cortex with the frontal component of the default mode network

The previous results suggest that SNS is related to variation in specific GM and WM structures, within an interconnected fronto-temporal and subcortical network in humans. However these methods cannot tell us whether these structural changes co-occur with functional differences within the network. We therefore complement these results by using rsfMRI to test whether the *functional interactions* between brain areas are also related to SNS. Using dual regression, we tested the functional contribution of brain regions to large distributed cortical networks [20,51].

Again motivated by previous work in the macaque [20], we focused on default mode network, a prominent resting state network, argued to reflect the default mode of social animals’ brain function, i.e. that of coordinating behaviour within a social context, a function more in demand in larger social networks [59]. Notably, there is substantial overlap between the DMN and the pattern of brain activity observed during tasks that tap into aspects of social cognition [20]. In macaques, the ACC is more functionally coupled with the (DMN) in animals living in larger social groups. We therefore tested whether the ACC was also coupled with the DMN in humans as a function of SNS.

**Fig. 4:**
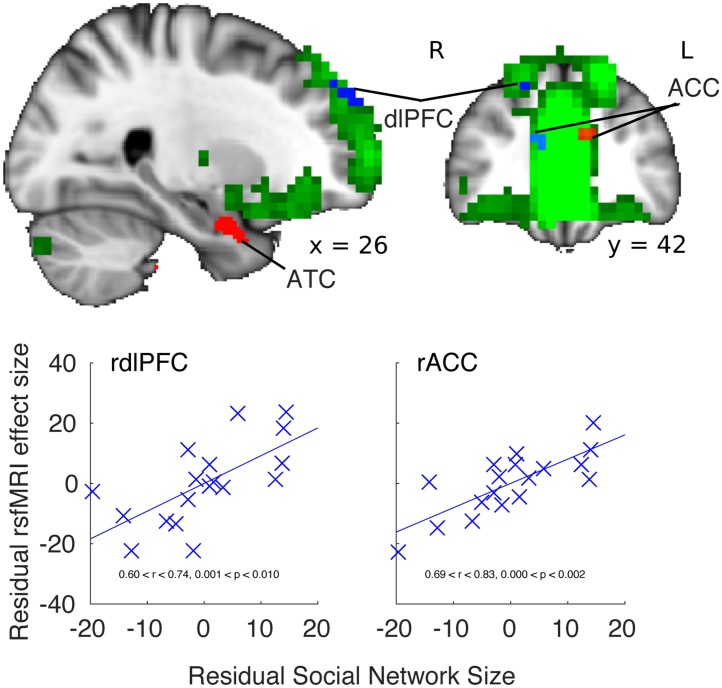
Dual regression results showing regions (blue, SVC p < 0.05) that are increasingly recruited into the aDMN (green) when subjects report larger social networks. VBM results are shown on slices for reference (red). Scatter plots illustrate the residual effect sizes against the residual of SNS taken from ROIs (64 mm^3^) placed at the centre of gravity of significant clusters, after accounting for age, gender and years of education. Images are in radiological convention. Abbreviations: dlPFC dorsolateral prefrontal cortex, ACC anterior cingulate cortex, ATC anterior temporal cortex. (For interpretation of the references to colour in this figure legend, the reader is referred to the web version of this article).

As in other work [60,61], here, ICA isolated the DMN as two independent components. We focused on the anterior component of the DMN (aDMN) which consisted of ventromedial prefrontal, cingulate cortex (mid and posterior), temporal pole, dorsolateral prefrontal cortex (dlPFC), striatum, thalamus, and hippocampus. Constraining the analysis to the aDMN component, an exploratory analysis identified the rostral dlPFC (Fig. 4; MNI coordinates of centre of gravity 26, 58, 34), bordering area 46 and 9 [62], as having significantly greater aDMN coupling as a function of SNS (small volume cluster-based thresholding corrected for multiple comparisons across the DMN component, with cluster-based thresholding c = 3.1, p < 0.05). By contrast, the equivalent analysis performed in control RSNs, or the pDMN failed to reach corrected significance (p > 0.05). Neither the sensory-motor, nor left or right dorsal attention stream contained regions for which coupling with the RSN varied as a function of SNS.

Given the importance of the anterior *dorsomedial* frontal cortex as a node of the DMN [63] and as an area involved in social cognition [64], further supported by the present VBM analyses, we investigated whether functional coupling between ACC and the DMN varied with SNS. This was borne out with a small volume correction, using an anatomically derived ROI of areas 24a + b and 32, which revealed a small left lateralised ACC cluster (SVC, with cluster-based thresholding c = 3.1, p < 0.05, MNI 10 40 18 Fig. 4). The equivalent ROI in the right hemisphere revealed a complimentary ACC cluster which failed to cluster correct over spatial extent (peak p = 0.318, MNI −2 26 24). Collectively, this analysis dovetails with previous work in monkeys showing that the ACC is increasingly recruited into the DMN when animals live in larger groups [20].

Dual regression effects were not driven by the observed differences in GM volume; they remained robustly equivalent after each subject’s GM jacobian value, at each voxel, was included in the permutation analysis as an additional voxel-dependent confound regressor. The effects were also not driven by outliers. A leave-one-out analysis on the two clusters validated our effects with all correlations remaining significant (0.6 < r < 0.83). Finally, variance in total intracranial volume could not explain the effects as relationships remained significant when individuals’ intracranial volume was accounted for (rACC r = 0.76, p = 0.0002, ldlPFC r = 0.640, p = 0.004). Note that head movements were removed during fMRI pre-processing.

### 3.4 Integrating gray matter differences correlating with SNS with structural and functional networks

**Fig. 5:**
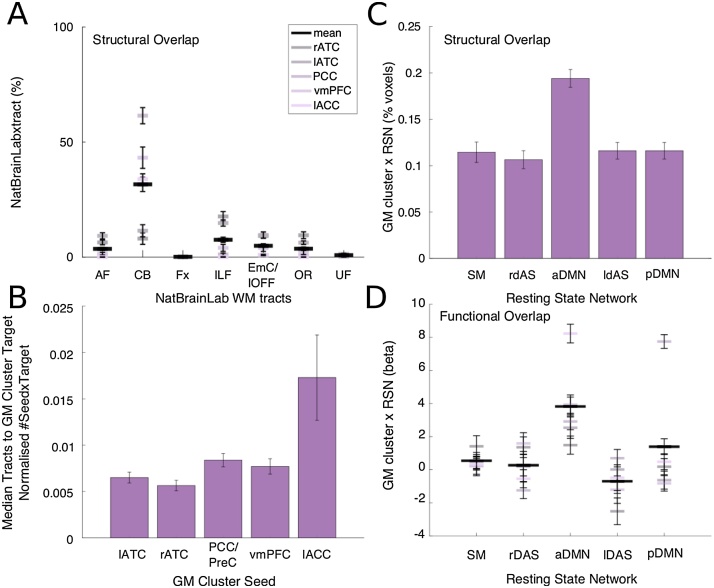
Network integration analyses. [A] Mean percentage overlap for each GM cluster seeded tractograms (grey-purple) and published WM tracts from the NatBrainLab. Black line plot shows mean percentage overlap over all three GM cluster seeded tractograms and between tractogram standard error. [B] Probabilistic tractography seeded in GM nodes with TBSS effects acting as waypoints and each non-seed defined as classification targets. Bar plot shows number of samples seeded from the GM cluster seeds reaching any target mask in the network. Subject’s median samples scores are normalised by the product of the size of the Seed and Target masks, then for each seed, averaged over targets and subjects [C] Mean percentage overlap between all GM clusters and each RSN. [D] Mean beta values expressing variance in the rsfMRI timeseries extracted from each GM cluster explained by each RSN timeseries. Black bars represents mean beta across the five GM clusters, and standard error averaged across GM clusters, for each RNS. Abbreviations: AF arcute fasciculus, CB cingulum bundle, Fx fornix, ILF inferior longitudinal fasciculus, EmC/lFOF extreme capsule / inferior fronto-occipital fasciculus, OR optic radiations, UF uncinate fasciculus, ATC anterior temporal cortex, PPC/PreC posterior parietal cortex / precuneus, ACC, anterior cingulate cortex, vmPFC ventromedial prefrontal cortex, SM sensory motor, dAS dorsal attention network, DMN default mode network. (For interpretation of the references to colour in this figure legend, the reader is referred to the web version of this article).

The analyses so far provide evidence for GM, WM and RSNs differences related to SNS. We next asked whether these findings were related. We tested whether the observed WM subregions, varying with SNS, form part of the pathways between the observed GM structures also varying with SNS. Each GM cluster identified by the VBM analysis (Fig. 3) was used as a seed in separate probabilistic tractography analyses. We constrained the tractography analysis to include only tracts that coursed through the TBSS effects Fig. 2A and calculated the percentage of spatial overlap between these GM cluster seeded tractograms and the predefined WM tracts from the NatBrainLab. Consistent with the tractography seeded directly from the WM sections (Fig. 2B), tractography seeded from the GM clusters in lACC, PCC/PreC and vmPFC all had pathways that heavily utilized the CB (Fig. 5A). Tractography seeded in the lATC and rATC coursed through the ILF and EmC/IFOF. The UF and ORs were also utilised by samples emanating from the ATC. A 7 (Tract) x 5 (GM cluster) repeated measures ANOVA confirms the degree of overlap varies across the predefined WM tracts (Tract: F_6,96_ = 181.93, p < 0.001), with some GM clusters, more than others, utilizing a distributed set of tracts (GM cluster: F_4,64_ = 12.13, p < 0.001). The significant interaction in the analysis (F_24,384_ = 50.01, p < 0.001) can be interpreted with post hoc comparisons showing the mean percentage overlap across all GM clusters (black line) differs significantly between all tracts (17.34 > t_16_ > 2.49, p < 0.024 with the exception between the UF and OR), suggesting that all GM clusters utilise the CB more than any other tract.

We then assessed the prominence of each GM node within the network. As a follow-up to the above probabilistic tractography analysis, the four non-seed regions now also acted as targets. The median number of samples connecting the seed and target was estimated for each subject. For each seeded tractography, this connectivity score was then averaged across all targets. Fig. 5B represents this global connectivity score from each seed to every target (e.g. ACC to (rATC + lATC + PCC + vmPFC)/4), averaged across subjects. A one-way repeated measures ANOVA showed a significant difference among the sample hit rates issued from the different seeds (F_4,64_ = 4.96, p = 0.031). Post hoc tests confirmed that the number of samples that successfully connected with one or other of the targets issued from ACC was significantly greater than from the ATC (ACC vs lATC: t_16_ = 2.45, p = 0.026, ACC vs rATC: t_16_ = 2.44, p = 0.027). To note, one subject’s samples emanating from ACC was greater than 3 standard deviations from the mean. When this subject is removed from the analyses, all between-seed post hoc comparisons are significant (ACC vs PCC: t_15_ = 2.24, p = 0.041, ACC vs vmPFC: t_15_ = 2.34, p = 0.034). This analysis contributes evidence that the ACC acts as a hub within the social brain.

Finally, we examined the structural and functional overlap of the VBM SNS effects and the DMN (Fig. 4 green cluster). First, we calculated percentage spatial overlap between the five social GM clusters and each individual’s aDMN, pDMN and three control RSNs (SM, rDAS and lDAS), normalised by component size (Fig. 5C). There were significant differences between overlap extent across the five RSNs (F_4,68_ = 15.7, p < 0.001). Crucially, the GM overlap is significantly greater with the aDMN than any other control network (all t_17_ > 4.94, p < 0.001).

Second, for each subject we independently regressed the raw resting-state timecourses from the five social GM clusters against the timecourses of our RSN (aDMN, pDMN, SM, rDAS, lDAS). As the RSNs compete to explain the variance in the VBM ROI timecourse we can directly compare the beta in a 5 (GM cluster) x 5 (RNS) repeated measures ANOVA (Fig. 5D). The variance explained by each RSN differs across and between GM clusters (GM cluster: F_4,68_ = 18.74, p < 0.001, RSN: F_4,68_ = 21.53 p < 0.001, Interaction: F_16,272_ = 14.34, p < 0.001). Post-hoc follow up statistics on the GM cluster average beta values suggest that only SM (t_17_ = 2.39, p = 0.047), aDMN (t_17_ = 11.26, p < 0.001) and pDMN (t_17_ = 5.32, p < 0.001) explain significant variance in the GM clusters’ timeseries. Further we show that the functional connectivity between social GM areas and the aDMN is significantly greater than the other RSNs with the aDMN time series explaining significantly more variance than all other RSN (all t_17 _> 4.28, p ≤ 0.001).

## 4 Discussion

**Fig. 6:**
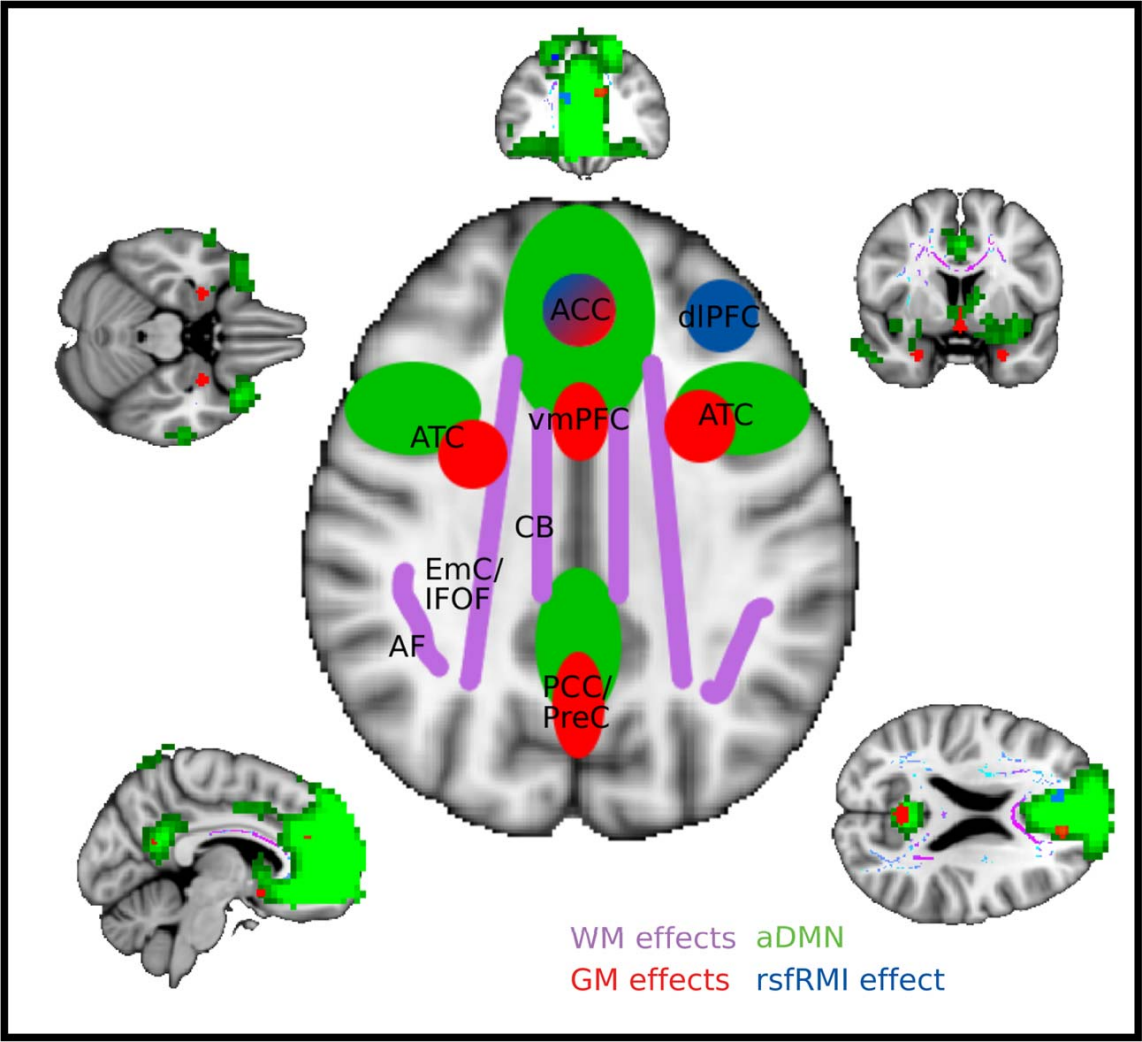
Schematic summary of the brain differences related to SNS. Key white matter (WM) tracts where fractional anisotropy varies as a function of SNS are illustrated in purple. Gray matter (GM) regions correlating with SNS are shown in red. Regions showing increased rsfMRI functional coupling with the anterior component of the default mode network (green) as a function of SNS. Abbreviations: AF arcute fasciculus, CB cingulum bundle, EmC/lFOF extreme capsule / inferior fronto-occipital fasciculus, dlPFC dorsolateral prefrontal cortex, ACC anterior cingulate cortex, ATC anterior temporal cortex, vmPFC ventromedial prefrontal cortex, PPC/PreC posterior parietal cortex / precuneus. (For interpretation of the references to colour in this figure legend and text, the reader is referred to the web version of this article).

The goal of this study was to investigate the relationship between brain organization and SNS. We first used DMRI to identify WM where fractional anisotropy correlated with SNS. Neuronal pathways connecting the frontal and temporal cortex, including the extreme capsule, inferior longitudinal fasciculus, cingulum bundle and arcuate fasciculus, show increased structural integrity in relation to larger SNS, as does the corpus callosum. In the same subjects, we then undertook a series of analyses to corroborate these findings and investigate the network interactions related to SNS. Using structural MRI, we identified GM nodes where volume was positively correlated with SNS. Macaque tracing and imaging studies have suggested that these GM regions are connected by fronto-temporal WM pathways including EmC, ILF, CB and AF [34,40]. We used probabilistic tractography in our human sample to confirm that the WM sections identified with TBSS were routes for the fibres emanating from these GM nodes and ultimately connected the network. Finally, using rsfMRI, we show that relative coupling of ACC and dlPFC (prominent targets of these WM tracts) with the anterior part of the default mode network (spatially contiguous with our social GM clusters) increases as a function of SNS. Fig. 6 summarises the results and demonstrates how the effects are related to each other.

In examining the relationship between social behaviour and brain organisation from a network perspective, our study had two key strengths. First, we were able to validate our findings with converging evidence from three complementary imaging techniques. The relative strengths and weaknesses of imaging methodologies are well documented, with each tuned to interrogate particular aspects of brain organisation. Across the imaging analyses, a distinct frontotemporal network was consistently evident. When applied together, these techniques build a case for the structural and functional brain basis of SNS and create a multi-modal map of a coherent network involved in the socio-cognitive behaviours tapped by living in social networks.

Second, our hypotheses were driven primarily by previous findings in macaque monkeys living in social groups of different sizes. Using very similar methods and analyses, we replicate in humans many of the core macaque effects that showed GM changes in the mPFC (including ACC) and amygdala, and increased functional coupling of the ACC with the DMN dependent on SNS [19,20]. While the human results do not speak to the direction of the relationship, SNS was under experimenter control in the macaque study, establishing that changes in SNS led to structural and functional brain changes. While we cannot exclude a contribution of pre-existing brain differences to SNS in humans, the macaque work suggests that the patterns we observed reflect the effects of social experience on the brain [50,62].

We used SNS indices as independent variables, as SNS constitutes a well-validated and characterised measure of social behavior [21,31]. It is notable that we find a relatively small number of brain regions connected by specific WM pathways related to this rather broad measure of social experience. Previous studies using this or related measures of SNS (online, 7 or 30 days social network index, or Norbeck Social Support Network) support key elements of the present work. Correlations with GM volume are described in sub-regions of temporal cortex, including entorhinal cortex and amygdala [[22], [23], [24]]. Furthermore, BOLD activity in these regions, measured while subjects made social closeness judgements, also correlates with individuals’ SNS [24]. While there appears to be slight variation in regional GM correlates of SNS, there is a core set of brain regions linked to SNS that seems robust to the choice of measure [24].

The socio-cognitive network described here can be speculatively related to three broad component processes: [1] valuation of the outcome of self and others’ choices, [2] mentalizing, and [3] social and emotion stimulus recognition. For example, the ACC and amygdala encode the value of social outcomes and affective stimuli respectively [[65], [66], [67]], and differences in the CB and EmC may reflect increased inter-regional neural transmission [34]. Alternatively, but not exclusively, our effects could reflect increased mentalizing abilities. Mentalizing skills correlates with SNS [21] and in line with previous literature, we observed GM differences in three prominent nodes of the DMN (ACC, PCC/PreC and temporal pole (within ATC cluster) [63]). Indeed, differences in functional couplings with the DMN and FA in the three long-range fronto-temporal WM pathways may be similarly attributable to mentalising [34]. For example, CB connects prominent nodes of the DMN [68], while anatomically the EmC in humans might reach all the way to the posterior part of the TPJ [57]. Relatedly, GM in these regions also correlates with empathy, a multi-faceted ability that allows us to share emotions with others [[69], [70], [71], [72], [73]]. Finally, individuals in larger social networks may also engage more frequently in higher-level facial and emotional processing and thus up-regulate another specialised network in the temporal cortex [[74], [75], [76], [77]] and medial prefrontal cortex [72,78,79]. Anterior temporal GM volume, and EmC and ILF FA differences may reflect this pressure [34].

Bridging the hemispheres, CC structural integrity also correlates with SNS. This prominent relationship is worthy of further study as disparate conclusions have been drawn on the role of this structure in social behavior [[80], [81], [82]]. A recent paper supports the present FA effects in a different and larger sample (n = 155, age 30–50) [25]. The authors emphasise the relationship between social diversity and WM integrity in the CC, but the large WM cluster they report also overlaps with CB and hypothalamic pathways. No other results were apparent, except at very lenient thresholds, perhaps because the analysis did not include a skeletonization step that corrects for misalignment of tracts between individuals, and thus affecting regional statistical power [35]. In contrast, we report significant whole-brain corrected results along specific sets of interconnected areas in temporal and frontal cortex.

Understanding normal social cognition from a network perspective could provide insight into the multifaceted symptoms presented by patients with social behavioral impairments. While some regions may play more critical roles in coordinating social behavior [7], multifocal or diffuse injury to this putative network may be particularly disabling [83,84]. Some psychiatric disorders also feature prominent social difficulties and such conditions may also be related to dysfunction at the network level [85,86]. Indeed, many psychiatric illnesses are associated with atypical connectivity of the DMN and nodes of the social brain [[87], [88], [89]]. As we have attempted here, understanding the contribution of the frontotemporal white matter tracts in social cognition will be essential to understanding the brain mechanisms underlying patients’ social impairments.

Reliability is an important consideration, as in any study. Sample sizes in the range of the current study have a reasonable track record in this regard [90], even for parametric designs [e.g. 48] and planned follow-up behavioural correlations [e.g. 65]. Despite the relatively small sample, the multi-modal approach allowed us to show between-methods consistency of network effects, while within-analysis tests of reliability confirmed that individual outliers did not drive the effects. Further, our results are particularly compelling when viewed together with the causal SNS effects on the brain detected with similar analyses in monkeys [19,20].

While our sample is relatively small it has a diverse make-up. Prior human studies have largely examined young adults (see Molesworth et al. [25], for an exception), while our sample included individuals ranging in age from 27 to 79 years. Older adults, with decades of social experience, form more stable social networks of better quality [91]. Thus, we believe this sample is a strength of the study, with the likely more stable social networks perhaps reducing the intra-subject signal-to-noise ratio in the imaging data.

In the current experimental design, we utilised a multimodal network analysis to focus on ‘depth’ over ‘breadth’, investigating the social brain network via three complementary imaging techniques and using this power to demonstrate the cross-modal reliability of the findings. The cingulate effects illustrate this point. We show [1] the structural integrity of the CB, [2] the GM volume of ACC and PCC, and [3] the functional coupling between the ACC and the aDMN, all correlate with SNS. Further, probabilistic tractography also suggested that the ACC is a prominent hub within the social brain.

In summary, we report a relationship between SNS and whole-brain anatomical networks. The findings emphasise the role of WM tracts in underpinning complex behaviors and highlight the advantages of using the multimodal approach to investigate brain organisation at a network level. Future studies should seek to develop our understanding of the fiber pathways identified here, as well as their relationship to the DMN. Finally, our results underline the neural complexities supporting social-group living and provide a principled starting point for investigating clinical social impairments from a network perspective.

## Competing Interests

The authors have no financial or non-financial competing interests to declare.
